# Supplementary material for: Diffusion-Weighted MRI for Predicting Pathologic Complete Response in Neoadjuvant Immunotherapy
Source: Cancers (Basel). 2022 Sep 13;14(18):4436. doi: 10.3390/cancers14184436 (PMC9497087; doi:10.3390/cancers14184436)
Supplement: Supplementary file 1 [file cancers-14-04436-s001.zip › cancers-1882378-supplementary.pdf]

Table S1. tumor ADC metrics and FTV values at T0.

MRI variable	Full (n=103)		Control (n=75)		Pembro (n=28)	
	**pCR (n=30)**	**Non-pCR (n=73)**	**pCR (n=15)**	**Non-pCR (n=60)**	**pCR (n=15)**	**Non-pCR (n=13)**
ADC_mean	0.92 (0.88, 0.99)	0.93 (0.86, 0.10)	0.92 (0.87, 0.99)	0.92 (0.86, 1.0)	0.92 (0.88, 0.99)	0.93 (0.88, 1.01)
ADC_min	0.64 (0.59, 0.72)	0.66 (0.57, 0.73)	0.65 (0.59, 0.70)	0.66 (0.57, 0.74)	0.62 (0.61, 0.72)	0.63 (0.57, 0.67)
ADC_5	0.73 (0.71, 0.79)	0.73 (0.65, 0.80)	0.74 (0.71, 0.81)	0.73 (0.65, 0.80)	0.72 (0.71, 0.78)	0.73 (0.65, 0.83)
ADC_15	0.80 (0.76, 0.85)	0.81 (0.72, 0.85)	0.82 (0.78, 0.87)	0.81 (0.71, 0.85)	0.77 (0.76, 0.83)	0.80 (0.74, 0.89)
ADC_25	0.84 (0.80, 0.89)	0.85 (0.76, 0.91)	0.85 (0.80, 0.91)	0.85 (0.76, 0.90)	0.82 (0.80, 0.88)	0.82 (0.80, 0.92)
ADC_50	0.91 (0.87, 0.99)	0.92 (0.84, 0.10)	0.92 (0.87, 0.99)	0.92 (0.84, 1.0)	0.90 (0.87, 0.99)	0.92 (0.89, 1.01)
ADC_75	0.98 (0.94, 0.11)	0.10 (0.94, 0.11)	0.99 (0.94, 1.06)	1.0 (0.94, 1.07)	0.98 (0.94, 1.07)	1.01 (0.96, 1.09)
ADC_95	0.11 (0.10, 0.12)	0.11 (0.10, 0.12)	1.09 (1.03, 1.18)	1.13 (1.04, 1.20)	1.09 (1.05, 1.21)	1.15 (1.05, 1.20)
ADC_max	0.13 (0.12, 0.14)	0.127 (0.115, 0.135)	1.27 (1.20, 1.38)	1.26 (1.15, 1.33)	1.25 (1.17, 1.42)	1.35 (1.20, 1.45)
FTV	15.23 (7.72, 32.36)	16.81 (7.93, 29.83)	16.53 (9.09, 32.26)	14.32 (7.55, 27.05)	12.48 (6.65, 27.18)	23.0 (16.16, 46.26)

Table S2. tumor ADC metrics and FTV values at T1.

MRI variable	Full (n=103)		Control (n=75)		Pembro (n=28)	
	**pCR (n=30)**	**Non-pCR (n=73)**	**pCR (n=15)**	**Non-pCR (n=60)**	**pCR (n=15)**	**Non-pCR (n=13)**
ADC_mean	1.08 (0.97, 1.17)	1.02 (0.92, 1.12)	1.11 (0.97, 1.16)	1.02 (0.93, 1.13)	1.06 (0.98, 1.17)	1.04 (0.92, 1.10)
ADC_min	0.83 (0.74, 1.01)	0.75 (0.61, 0.87)	0.84 (0.75, 0.93)	0.75 (0.63, 0.87)	0.80 (0.71, 1.04)	0.69 (0.59, 0.83)
ADC_5	0.90 (0.78, 1.02)	0.80 (0.69, 0.90)	0.92 (0.79, 1.0)	0.81 (0.69, 0.90)	0.89 (0.77, 1.06)	0.74 (0.69, 0.87)
ADC_15	0.94 (0.80, 1.05)	0.87 (0.78, 0.97)	0.96 (0.86, 1.05)	0.88 (0.78, 0.98)	0.93 (0.77, 1.06)	0.81 (0.78, 0.96)
ADC_25	0.99 (0.89, 1.10)	0.91 (0.83, 1.01)	1.03 (0.90, 1.09)	0.92 (0.83, 1.02)	0.98 (0.87, 1.12)	0.88 (0.83, 0.90)
ADC_50	1.07 (0.97, 1.13)	1.01 (0.91, 1.11)	1.11 (0.97, 1.13)	1.01 (0.93, 1.11)	1.05 (0.97, 1.14)	1.03 (0.91, 1.12)
ADC_75	1.14 (1.03, 1.22)	1.08 (1.0, 1.19)	1.17 (1.03, 1.24)	1.08 (1.0, 1.19)	1.12 (1.03, 1.19)	1.08 (0.99, 1.19)
ADC_95	1.23 (1.15, 1.33)	1.20 (1.10, 1.29)	1.26 (1.21, 1.36)	1.20 (1.10, 1.30)	1.21 (1.13, 1.23)	1.18 (1.13, 1.27)
ADC_max	1.32 (1.25, 1.44)	1.33 (1.18, 1.45)	1.37 (1.27, 1.49)	1.32 (1.17, 1.45)	1.28 (1.24, 1.33)	1.40 (1.22, 1.45)
FTV	3.95 (1.58, 20.66)	9.14 (4.62, 29.32)	4.23 (1.18, 24.40)	8.07 (4.37, 28.64)	3.67 (1.67, 11.76)	11.69 (8.17, 42.10)

Unit of ADC values: 10^{-3} mm^2/s. Unit of FTV values: cubic centimeter. Values are presented as median (interquartile range).

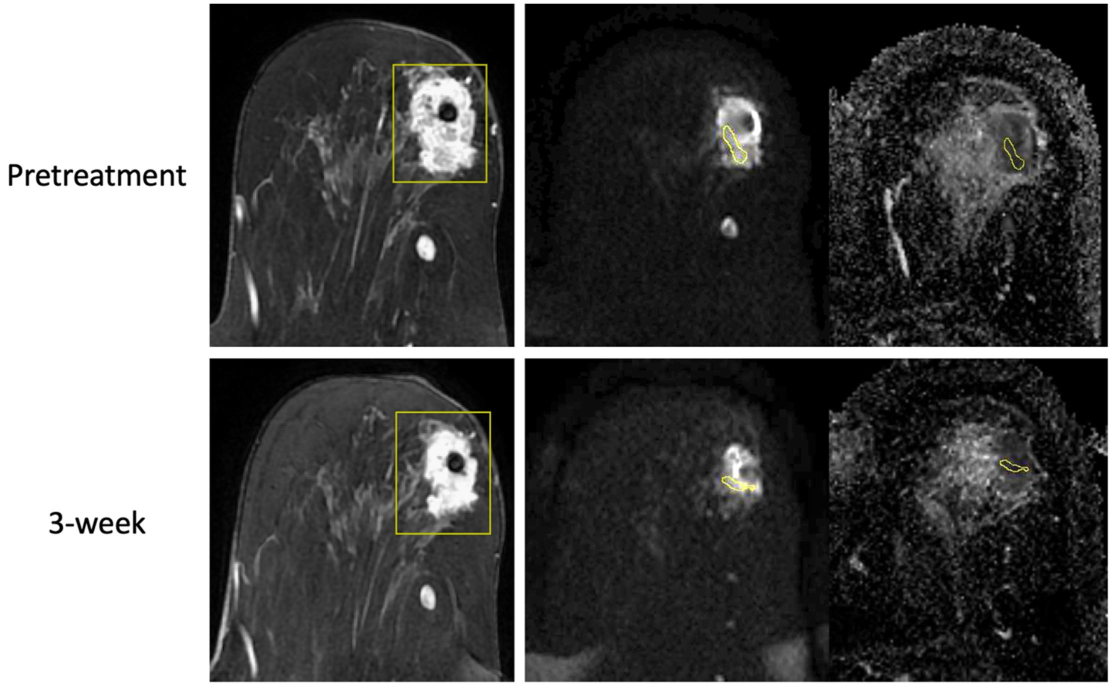

Figure S1. Example case 3 with a pathologic complete response. The patient was treated by paclitaxel followed by cyclophosphamide. Representative MR images are shown from pretreatment (top row) and 3-week (bottom row). Images from DCE-MRI were acquired 130 s after the contrast injection. Images from DWI are shown in a pair of original DWI (b = 800 s/mm^2) and ADC map. ROIs are shown in yellow (rectangular box in DCE and hand-drawn in DWI). The tumor FTV was 18.1 cc at T0 and 7.6 cc at T1 (58% decrease). ADC_mean was 0.921 × 10^{-3} mm^2/s at T0 and 1.112 × 10^{-3} mm^2/s at T1 (21% increase).

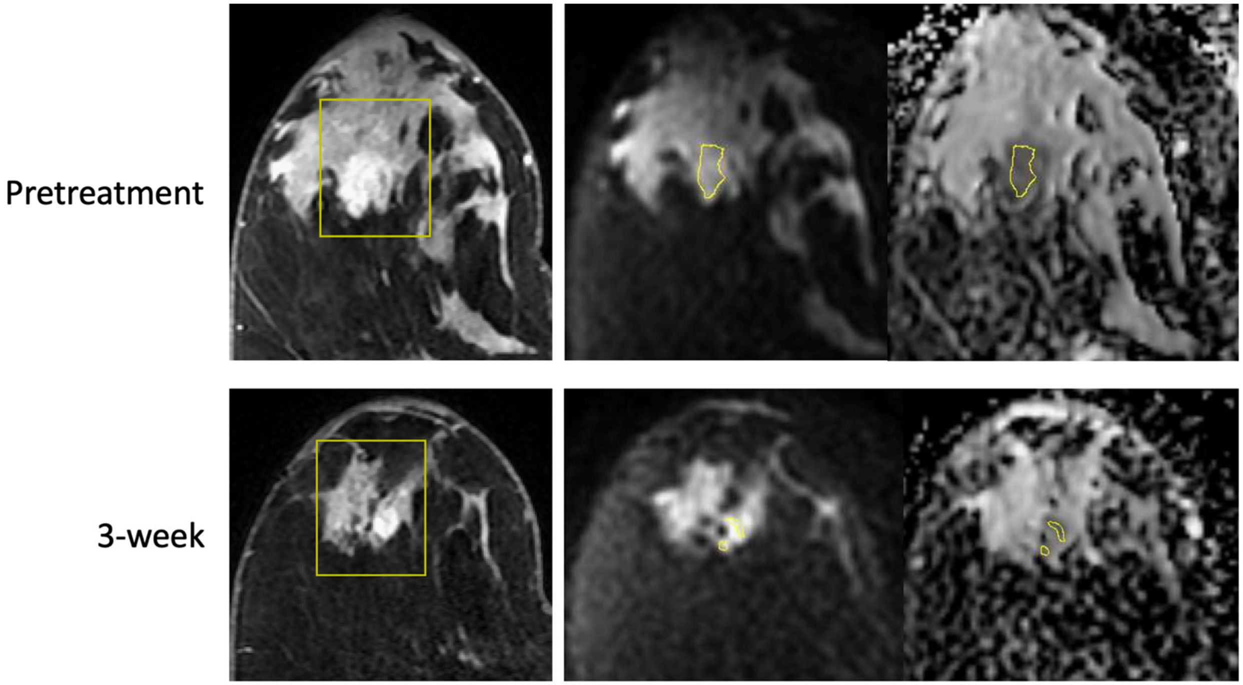

Figure S2. Example case 4 without a pathologic complete response. The patient was treated by paclitaxel followed by cyclophosphamide. Representative MR images are shown from pretreatment (top row) and 3-week (bottom row). Images from DCE-MRI were acquired 129 s after the contrast injection. Images from DWI are shown in a pair of original DWI (b = 800 s/mm^2) and ADC map. ROIs are shown in yellow (rectangular box in DCE and hand-drawn in DWI). The tumor FTV was 9.3 cc at T0 and 1.3 cc at T1 (85% decrease). ADC_mean was 1.111 × 10^{-3} mm^2/s at T0 and 0.942 × 10^{-3} mm^2/s at T1 (15% decrease).
